# Cryo‐EM structure of GH43 β‐Xylosidase from *Enterobacter cloacae* provides insights into substrate specificity and the role of an auxiliary domain in enzymatic activity

**DOI:** 10.1111/febs.70590

**Published:** 2026-05-18

**Authors:** Lorenzo Briganti, Andre Schutzer Godoy, Caio Cesar Mello Capetti, Rafaela Sachetto Fernandes, Vanessa O. A. Pellegrini, Ornella M. Ontañon, Eleonora Campos, Rodrigo V. Portugal, Igor Polikarpov

**Affiliations:** ^1^ São Carlos Institute of Physics University of São Paulo Brazil; ^2^ Echo Life Sciences São Carlos Brazil; ^3^ Instituto de Agrobiotecnología y Biología Molecular (IABIMO), CONICET‐INTA Prov. Buenos Aires Argentina; ^4^ Brazilian Nanotechnology National Laboratory CNPEM Campinas São Paulo Brazil

**Keywords:** Cryo‐EM, EcXyl43, Enterobacter, GH43, xylan, β‐xylosidase

## Abstract

Xylan is the second most abundant polysaccharide in plant cell walls, consisting of a β‐1,4‐linked xylopyranosyl backbone that can be substituted with various side chains. Depolymerization of xylan is predominantly catalyzed by the coordinated activity of β‐xylanases and β‐xylosidases. In this study, we determined the cryo‐electron microscopy (cryo‐EM) structure of *Enterobacter cloacae* β‐xylosidase (*Ec*Xyl43), a glycoside hydrolase family 43 (GH43) enzyme. Additionally, we resolved the X‐ray crystal structure of a catalytically inactive F507A mutant of EcXyl43. Together, these structures represent the first structural characterization of a β‐xylosidase from the *Enterobacter* genus using both X‐ray diffraction and cryoEM. Furthermore, to elucidate the molecular basis of substrate recognition and specificity, we conducted molecular dynamics simulations of the enzyme. Structural and computational analysis of *Ec*Xyl43 identified key determinants of the enzyme's preference for longer xylooligosaccharides and revealed how noncatalytic residues within the auxiliary domain modulate its activity.

## Graphical abstract

Xylan, the second most abundant plant cell wall polysaccharide, is degraded by β‐xylanases and β‐xylosidases. Here, we present the 2.65 Å cryo‐EM structure of *Enterobacter cloacae* β‐xylosidase (EcXyl43, GH43) and the 2.4 Å X‐ray structure of its inactive F507A mutant. Combined structural and molecular dynamics analyses reveal determinants of substrate specificity, highlighting how auxiliary‐domain residues regulate recognition of longer xylooligosaccharides and modulate catalytic activity.

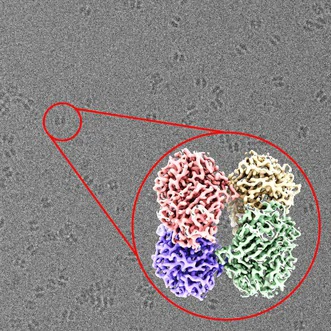

**Abbreviations**
Å: ångström
BLASTp: Basic Local Alignment Search Tool for proteins
CAZy: Carbohydrate‐Active enZymes database
CBM: carbohydrate‐binding module
CC: correlation coefficient
cryo‐EM: cryo‐electron microscopy
CTF: contrast transfer function
DALI: Distance‐matrix ALIgnment
DMSO: dimethyl sulfoxide
EcXyl43: Enterobacter cloacae glycoside hydrolase family 43 β‐xylosidase
EMDB: Electron Microscopy Data Bank
EMPIAR: Electron Microscopy Public Image Archive
FSC: Fourier shell correlation
GH: glycoside hydrolase
GH43: glycoside hydrolase family 43
GsXyl: Geobacillus stearothermophilus β‐xylosidase
IPTG: isopropyl β‐D‐thiogalactopyranoside
kDa: kilodalton
LB: Luria–Bertani medium
MD: molecular dynamics
MX: macromolecular crystallography
NADPH: nicotinamide adenine dinucleotide phosphate (reduced form)
Ni‐NTA: nickel‐nitrilotriacetic acid
OD600: optical density at 600 nm
PDB: Protein Data Bank
pNP: p‐nitrophenol
pNPX: 4‐nitrophenyl β‐D‐xylopyranoside
PPP: pentose phosphate pathway
Rfree: free R‐factor
RMSD: root‐mean‐square deviation
RMSF: root‐mean‐square fluctuation
Rpim: precision‐indicating merging R‐factor
SDS/PAGE: sodium dodecyl sulfate/polyacrylamide gel electrophoresis
SrXyl: Selenomonas ruminantium β‐xylosidase
WT: wild‐type
X2: xylobiose
X6: xylohexaose
XOS: xylooligosaccharides
α‐L‐AF: α‐L‐arabinofuranosidase

## Introduction

The Plantae kingdom plays a vital role in terrestrial ecosystems and is distinguished by remarkable diversity in plant forms, habitats, and physiological adaptations [1]. A defining feature of this kingdom is the presence of carbohydrate‐rich cell walls, which are essential throughout the plant life cycle [2]. These walls regulate cell size and shape, provide structural support for growth and development, serve as protective barriers, and act as sources of biologically active regulatory molecules [2]. Plant cell walls make up the majority of a plant's dry weight and are primarily composed of three major polymers: cellulose, hemicellulose, and lignin. On average, dry plant biomass consists of 40–50% cellulose, 15–25% hemicellulose, 20–25% lignin, and 5–10% other constituents [3]. Among these, hemicelluloses are the second most abundant class of plant polysaccharides, typically accounting for 10–30% of tissue dry weight, depending on the tissue type [4].

Xylan, the most prevalent form of hemicellulose, comprises a structurally diverse group of polysaccharides [5]. Its backbone consists of D‐xylose residues linked by β‐1,4‐glycosidic bonds [6], which can be further modified by (4‐O‐methyl)‐α‐1,2‐linked D‐glucuronic acid or decorated with α‐1,2‐ or α‐1,3‐linked D‐arabinofuranose residues. Additionally, O‐acetyl groups may be attached to the xylan backbone, further increasing its structural complexity and resistance to degradation [7].

Since hemicellulose is a highly heterogeneous group of biopolymers, its enzymatic degradation requires a complex array of enzymes [8]. This enzymatic system includes glycoside hydrolases (GHs, EC 3.2.1.‐) with diverse catalytic activities. Among them, endo‐1,4‐β‐D‐xylanases (EC3.2.1.8) cleave internal bonds within the xylan backbone, while 1,4‐β‐D‐xylosidases (EC3.2.1.37) act on xylooligosaccharides (XOS). Similarly, endo‐1,4‐β‐D‐mannanases (EC3.2.1.78) target internal linkages in mannans, whereas 1,4‐β‐D‐mannosidases (EC3.2.1.25) break down mannooligosaccharides into mannose. Various accessory enzymes facilitate side‐chain removal, including α‐D‐galactosidases (EC3.2.1.22), α‐L‐arabinofuranosidases (EC3.2.1.55), α‐glucuronidases (EC3.2.1.139), acetyl xylan esterases (EC3.1.1.72), and feruloyl/p‐coumaric acid esterases (EC3.1.1.73) [8, 9].

One of the most versatile glycoside hydrolase families involved in hemicellulose degradation is GH43, part of the 194 GH families classified in the CAZy database [10]. GH43 enzymes exhibit a range of hemicellulolytic activities, with the most common being β‐xylosidases [11], α‐L‐arabinofuranosidases [12], and endo‐α‐L‐arabinanases [13]. Recently, this family has been further divided into 40 subfamilies based on sequence and functional diversity [14]. GH43 β‐xylosidases catalyze the hydrolysis of short xylooligosaccharides (XOS) into xylose through an inverting mechanism, which prevents transglycosylation and the formation of longer oligosaccharides [15].

The genus *Enterobacter*, a member of the *Enterobacteriaceae* family, comprises Gram‐negative, facultatively anaerobic, rod‐shaped, and non‐spore‐forming bacteria [15]. *Enterobacter cloacae* is widely distributed in nature and holds clinical relevance as an opportunistic pathogen, especially in intensive care settings, due to its intrinsic resistance to multiple antibiotics [16]. To date, EcXyl43 is the only glycoside hydrolase family 43 (GH43) enzyme from an *Enterobacter* species that has been heterologously expressed and biochemically characterized [17, 18]. This enzyme belongs to subfamily 11 of the GH43 family. Unlike some GH43 β‐xylosidases—such as WXyn43 from the putative probiotic *Weissella* sp. strain 92, which shows highest activity on shorter XOS in the order X2 > X3 > X4 [19]—EcXyl43 exhibits greater catalytic efficiency on longer XOS and only weak activity on commercial xylan. Site‐directed mutagenesis studies have identified several key catalytic residues; specifically, substitutions D14A, W73G, and F507A were found to completely abolish enzymatic activity [17].

Once xylose is transported into the bacterial cytoplasm and isomerized to D‐xylulose, it enters the pentose phosphate pathway (PPP), the primary metabolic route for xylose utilization in most organisms [20, 21]. The PPP is conserved across all domains of life and plays a central role in cellular metabolism by generating D‐ribose for nucleic acid synthesis, D‐erythrose 4‐phosphate for the biosynthesis of aromatic amino acids, and NADPH for various anabolic processes [22]. Understanding the enzymatic machinery involved in xylose metabolism is therefore critical for elucidating how bacteria adapt to diverse environments and carbon sources.

This study aimed to investigate the structural determinants underlying the enhanced catalytic efficiency of *Enterobacter cloacae* GH43_11 β‐xylosidase toward longer‐chain XOS with degrees of polymerization up to six. To elucidate these mechanisms, we determined the first cryo‐electron microscopy (cryo‐EM) structure of the wild‐type (WT) GH43_11 β‐xylosidase, alongside the X‐ray crystallographic structure of a catalytically inactive mutant, EcXyl43 F507A. Molecular dynamics simulations were employed to further characterize substrate‐enzyme interactions and conformational dynamics in solution. In addition, we probed the putative functional roles of noncatalytic residues within the auxiliary domain, revealing their contribution to structural modulation and enzymatic activity.

## Results and Discussion

### Cloning, expression, purification, and enzymatic assays

*Ec*Xyl43 clone was transformed into *E. coli* Rosetta cells. *Ec*Xyl43 was expressed and purified using Ni^+2^ affinity chromatography. The theoretical mass of *Ec*Xyl43 predicted based on its amino acid sequence is 64.4 kDa and the purified protein migrated as a single band on SDS/PAGE, thus assuring its purity. The purified enzyme was used for the determination of its activity and for crystallization assays. In line with the previous results [17] and differently for *Ec*Xyl43, the *Ec*Xyl43 F507A mutant did not have any detectable enzymatic activity.

### Cryo‐EM data collection, processing, and structure refinement

Despite extensive efforts, we were unable to obtain crystals of WT EcXyl43. Therefore, we employed cryo‐EM single‐particle analysis to determine its structure (Fig. 1). The structure of WT EcXyl43 was determined using single‐particle cryo‐EM at 300 kV on a Falcon 3EC direct electron detector with a pixel size of 2.46 Å, and it is the first structure of its kind determined with this method. For that, a total of 10 176 movies were collected, with a total electron dose of 30 e^−^/Å^2^ fractionated into 20 frames (1.5 e^−^/Å^2^ per frame). The defocus range varied from −1.5 to −2.0 μm. Following particle picking and classification, 438 836 particles were used for refinement. The final reconstruction yielded a tetrameric structure with four chains and a total of 17 137 atoms across 2133 residues. The structural model exhibited excellent stereochemistry, with bond length and angle RMSD values of 0.005 Å and 0.728°, respectively. The MolProbity score was 2.03, with a low clash score of 6.59. The Ramachandran plot showed 95.19% of residues in favored regions, 4.53% in allowed regions, and only 0.28% as outliers. Rotamer outliers were minimal (2.74%), with no Cβ or peptide plane outliers (Table 1). The final map was refined to a global resolution of 2.64 Å (gsFSC 0.143), with local resolutions ranging from 2.2 to 3.2 Å. The model fit to the data was validated by cross‐correlation coefficients of 0.83 (masked) and 0.82 (box). The atomic coordinates and cryo‐EM map were deposited in the Protein Data Bank (PDB id: 8UWS) and the Electron Microscopy Data Bank (EMDB id: EMD‐42680), with raw movies available in EMPIAR (EMPIAR‐11768).

**Fig. 1:**
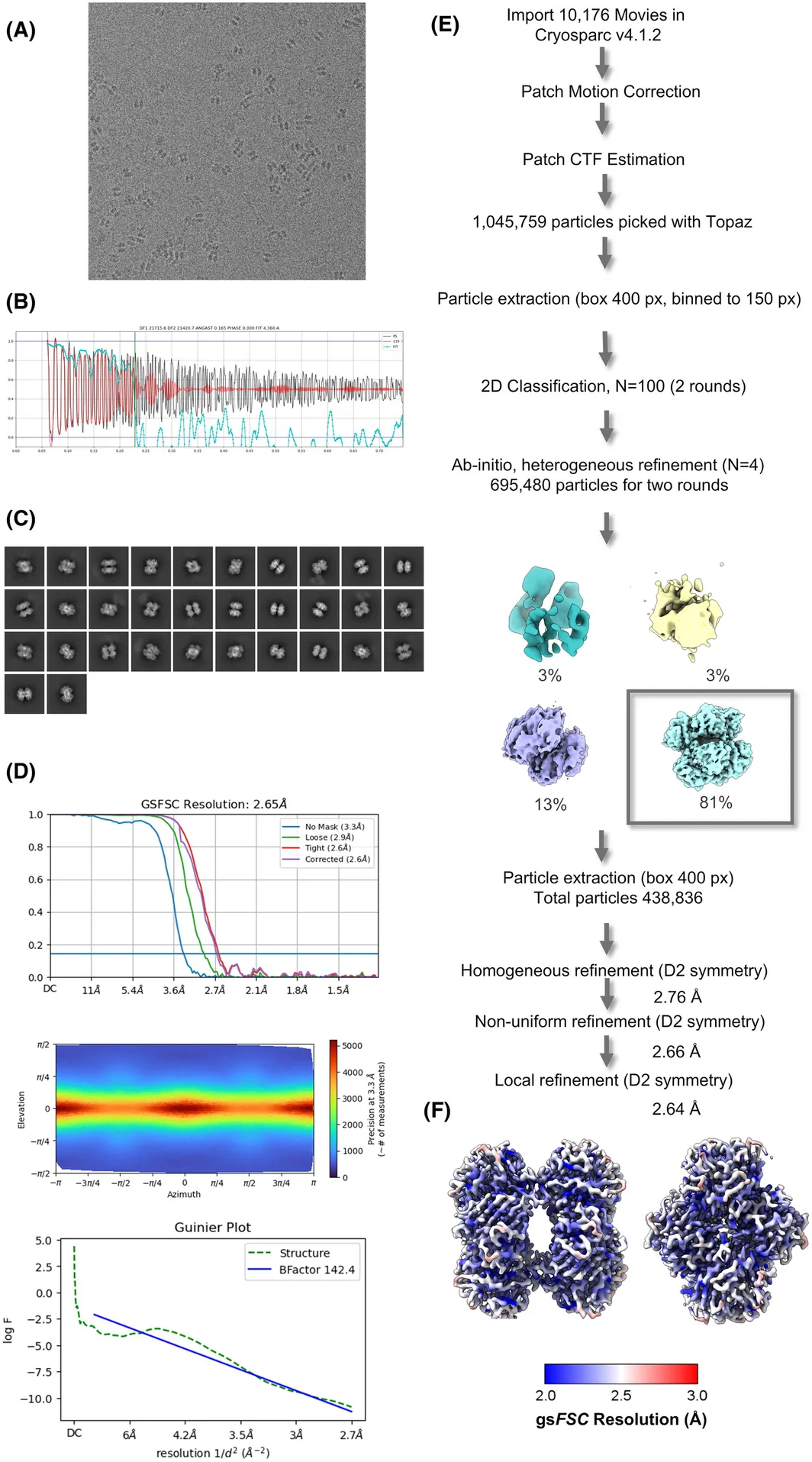
(A) Aligned micrographs. (B) CTF‐function calculated from obtained micrographs. (C) 2‐D classes obtained during data processing. (D) Fourier shell correlation (FSC) between half maps of the final reconstructions. At the top, graph shows FSCs versus resolution of masked and unmasked densities. At the middle, azimuth distribution of particles. At the bottom, Guinier plot showing calculated B‐factor. (E) Detailed schematic of steps taken for final reconstruction, highlighting obtained 2D and 3D classes, and first high‐resolution reconstruction. (F) Local resolution projected on the final map from two orientations. Figures were generated with Cryosparc and ChimeraX.

**Table 1:** *Ec*Xyl43 cryo‐EM data collection and model refinement statistics.

Data collection	
Voltage (keV)	300
Camera	Falcon 3EC
Pixel size (Å)	2.46
Total dose (e/Å2)	30
Fractions	20
Dose per frame (e/Å2/frame)	1.5
Defocus range (μm)	1.5 to 2
Movies collected	10 176
Data processing and Refinement	
Particles	438 836
Chains	4
Atoms	17 137
Residues	2133
Bonds (RMSD)	
Length (Å)	0.005
Angles (°)	0.728
MolProbity score	2.03
Clash score	6.59
Ramachandran plot (%)	
Outliers	0.28
Allowed	4.53
Favored	95.19
Rotamer outliers (%)	2.74
Cβ outliers (%)	0
Peptide plane (%)	0
CaBLAM outliers (%)	3.14
Box	
Lengths (Å)	110.55, 103.18, 113.9
Angles (°)	90, 90, 90
Masked dFSC model (0/0.143/0.5)	2.2, 2.64, 3.2
Model vs. Data	
CC (mask)	0.83
CC (box)	0.82
PDB code	8UWS
EMDB	EMD‐42680
EMPIAR	EMPIAR‐11768

### *Ec*Xyl43 structure and tetrameric assembly

The structure revealed a tetrameric assembly with D2 symmetry, resembling the GH43 β‐xylosidases from *Geobacillus stearothermophilus* (GsXyl43) and *Selenomonas ruminantium* (Fig. 2A) [23, 24]. The overall fold of each monomer EcXyl43 shares significant structural similarity with GsXyl43, with 57% sequence identity. Like GsXyl43, each EcXyl43 monomer consists of two domains connected by a flexible linker: an N‐terminal catalytic domain (Residues 1–320) and a C‐terminal β‐sandwich auxiliary domain (Residues 330–536) (Fig. 2B).

**Fig. 2:**
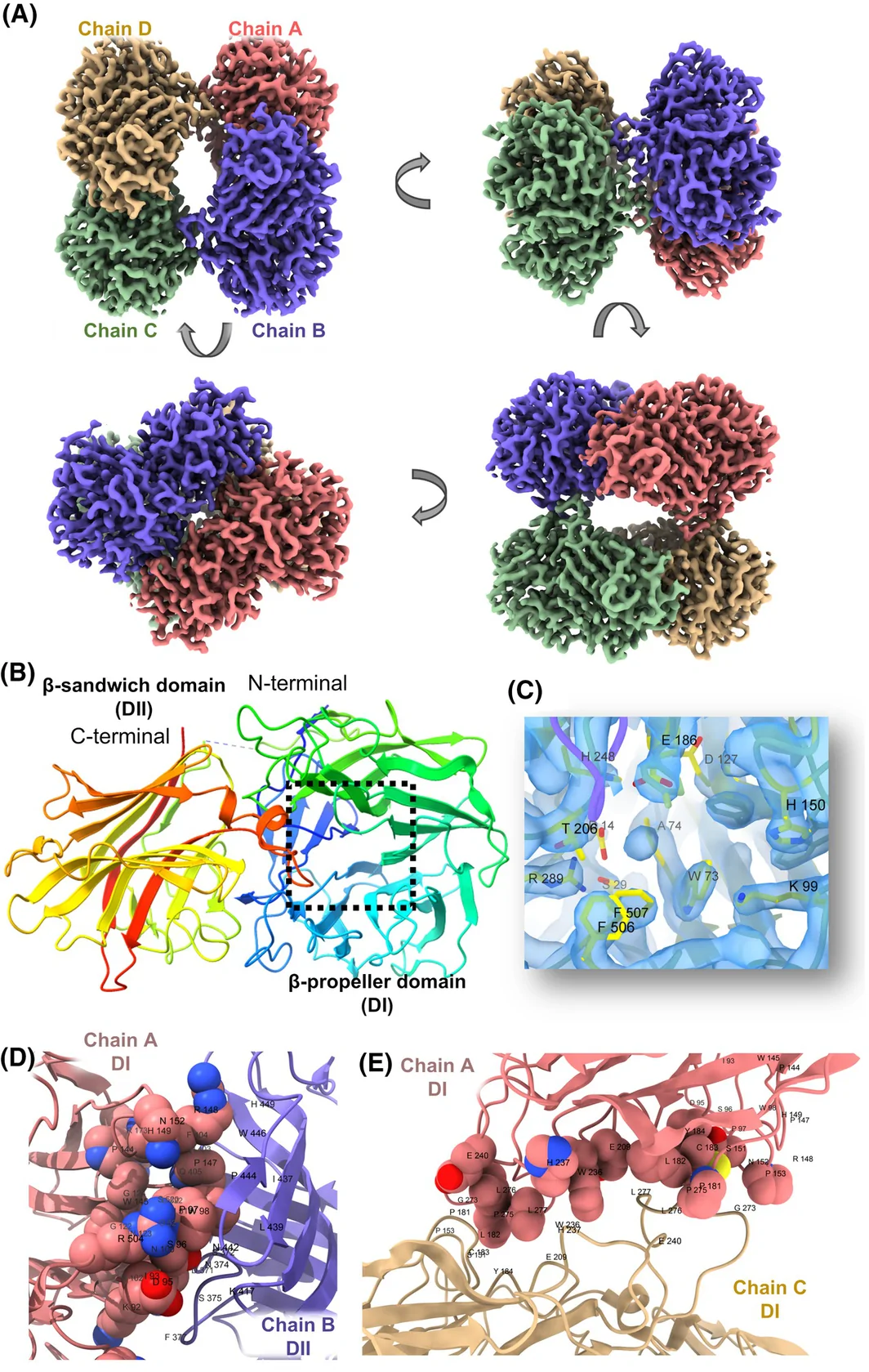
Structural insights of EcXyl43. (A) Rotated view of the cryo‐EM density map of the EcXyl43 tetrameric assembly. (B) Cartoon representation of a single EcXyl43 monomer, colored as a rainbow gradient from blue (N terminus) to red (C terminus). The location of the active site is highlighted by a dashed square. (C) Close‐up view of the catalytic site. Selected residues are shown as yellow sticks, and the corresponding cryo‐EM density is displayed as a blue volume contoured at 0.255σ. (D) Interface between chains A (salmon) and B (purple), showing the interactions between their respective domains I (DI) and II (DII), which generate the C2 symmetry of the tetramer. Contacting residues of DI are shown as spheres to demonstrate tight packing with DII. (E) Interface between chains A (salmon) and C (beige), involving interactions between their DI domains and giving rise to the overall D2 symmetry of the complex. Figures were generated with ChimeraX.

The catalytic domain adopts the characteristic five‐bladed β‐propeller fold typical of GH43 enzymes. Each blade is composed of four β‐strands arranged radially around the central axis of the propeller, forming a highly twisted topology. As observed for other GH43 members, such as α‐l‐arabinanase and levansucrase, this structural motif supports the hydrolytic activity of the enzyme (Fig. 2B). The quality of the cryo‐EM map supports the reliability of the structural interpretation (Fig. 2C).

The auxiliary β‐sandwich domain (residues 330–535) consists of two antiparallel β‐sheets, forming a stable immunoglobulin‐like fold. Structural comparison using DALI suggests that this domain bears resemblance to carbohydrate‐binding modules (CBMs), such as the lectin‐like domain of *Trypanosoma rangeli* sialidase (PDB: 1MZ5) [25]. However, while it may have evolved from a CBM, in EcXyl43, this domain primarily contributes to inter‐subunit interactions and stabilizes the tetrameric assembly. Additionally, a loop within this domain (Residues 502–510) extends into the active site of the enzyme and is positioned at the entrance of the catalytic tunnel. Its spatial location and orientation suggest that this loop may influence the geometry of the tunnel opening and consequently could affect substrate accessibility.

The tetrameric assembly of *E. coli* β‐xylosidase *Ec*Xyl43 consists of two tightly associated dimers, in which each monomer aligns in an antiparallel orientation relative to its dimeric partner. Similar to *Gs*Xyl43, the interface between the two monomers is formed by interactions between the N‐terminal β‐propeller domain of one monomer and the C‐terminal β‐sandwich domain of an adjacent monomer (Fig. 2D).

The monomer–monomer C2 symmetry interface is stabilized by hydrogen bonds between key conserved residues located in the loops extending from blades II and III of the β‐propeller domains (Fig. 2D). The dimer–dimer D2 symmetry interactions within the tetrameric structure involve three key loop regions from the β‐propeller domain (Fig. 2E). Similar tetrameric arrangements have been observed in homologous β‐xylosidases, such as those from *B. subtilis* (PDB id: 1YIF) and *Clostridium acetobutylicum* (PDB id: 1Y7B). The tetrameric structure of *Ec*Xyl43 suggests this cryo‐EM study represents the first direct evidence of a GH43 β‐xylosidase adopting this oligomeric state in solution (Fig. 2A). [24]. These structural insights into EcXyl43 provide a crucial foundation for understanding its enzymatic mechanism and potential functional divergence within the GH43 family.

### EcXyl43 F507A crystallographic structure determination and analysis

Although we were unable to crystalize WT EcXyl43, EcXyl43 F507A generated abundant well‐diffracting crystals. The crystal structure of the EcXyl43 F507A variant was determined at a resolution of 2.4 Å using X‐ray crystallography at the LNLS MX2 beamline. The crystals belonged to the orthorhombic space group *P*2_1_ 2_1_ 2_1_, with unit cell parameters *a* = 87.12 Å, *b* = 153.10 Å, and *c* = 182.40 Å. Data collection statistics indicated high completeness (99.74%) and a multiplicity of 13.1, with an overall *R*_pim_ of 0.075 and CC_1/2_ of 0.99.

Refinement terminated with *R*_work_ of 0.19 and an *R*_free_ of 0.22, with four molecules in the asymmetric unit, each containing 536 residues. The structure exhibited excellent geometry, with an overall MolProbity score of 1.48, a mean B‐factor of 42.10 Å^2^, and minimal outliers (0.42% Ramachandran outliers and 0% rotamer outliers). The final model included ligands such as glycerol (GOL) and calcium (CA), further supporting the structural integrity of EcXyl43 F507A. Both collection and refinement parameters are given in Table 2. EcXyl43 F507A crystal structure revealed a biological unit similar to the one observed for native in cryo‐EM experiments.

**Table 2:** *Ec*Xyl43 F507A crystallographic data collection and model refinement statistics. *R*_*pim*_ = ∑_h_ [1/(*n*_h_−1)]^1/2^ ∑_i_|<*I*_h_ > − *I*_h,i_|/∑_h_ ∑_I_ *I*_h,i_. *R*_*work*_ = ∑_hkl_(||F_obs_|−|F_calc_||)/∑_hkl_|F_obs_|, where |F_obs_| and |F_calc_| are the observed and calculated structure factor amplitudes, respectively.

Dataset	*Ec*Xyl43 F507A
Data collection	LNLS MX2
Wavelength (Å)	1.458
Space group	P 2_1_ 2_1_ 2_1_
Cell Parameters	
*a*, *b*, *c* (Å)	87.12153.10182.40
Resolution range (Å)	22.71–2.4 (2.486–2.4)*
*R*_*pim*_	0.075 (0.66)
CC_1/2_	0.99 (0.43)
Total reflections	1 253 638 (118376)
Unique reflections	95 858 (9404)
Multiplicity	13.1 (12.5)
Completeness (%)	99.74 (99.11)
< I/$\sigma_{I}$>	8.73 (1.13)
Refinement	
No. mol. asymmetric cell unit	4
Residues per molecule	536
*R*_work_	0.19 (0.29)
*R*_free_	0.22 (0.31)
Overall CC	0.998 (0.78)
Ligands	GOL, CA
B‐factors (Å^2^)	
Mean	42.10
Macromolecules	42.23
Ligands	43.09
R.m.s. deviations	
Bond lengths (Å)	0.003
Bond angles (°)	0.65
Ramachandran	
Ramachandran favored (%)	95.47
Ramachandran allowed (%)	4.11
Ramachandran outliers (%)	0.42
Rotamer outliers (%)	0
MolProbity overall score	1.48

* Values in parentheses are for highest‐resolution shell. Structure is based on single crystal.

### Structural comparison of EcXyl43 with homologous GH43 enzymes

To compare the structure of EcXyl43 with other GH43 enzymes, we performed a BLASTp search using its protein sequence and analyzed its crystallographic structure with DALI. This resulted in the identification of three homologous enzymes: *Gs*Xyl, *Sr*Xyl, and *Bp*Xyl (Table 3).

**Table 3:** Alignment parameters for the enzymes selected from BLASTp and DALI. The values obtained in comparison to *Ec*Xyl43.

Enzyme	PDB id	Identity (%)	Query cover (%)	R.M.S.D. (Å)
*Gs*Xyl	2EXH	56.06	99	1.2
*Sr*Xyl	3C2U	53.99	100	1.2
*Bp*Xyl	5ZQX	51.95	99	1.1

Multiple sequence alignments were conducted using Clustal Omega, followed by structure‐based sequence alignment with ESPript (Fig. 3). Structural superposition revealed a high degree of conservation, with notable differences in the loop spanning Residues 145–155 (EcXyl43 numbering) and the region involving Residues 502–510. These regions encompass the active site pocket and may be involved in structural rearrangements upon substrate binding.

**Fig. 3:**
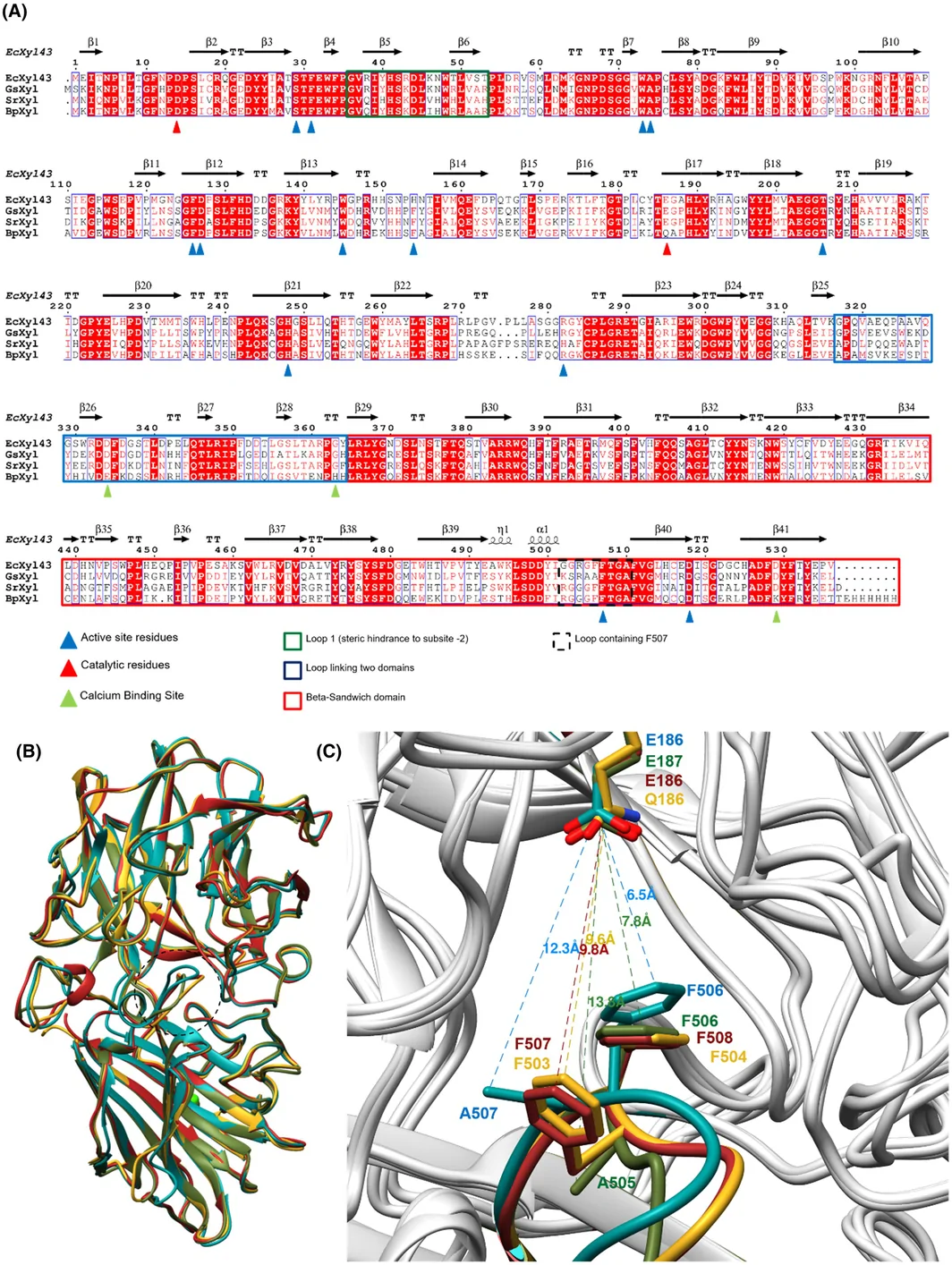
Structure‐based sequence alignment. (A) The sequences of *Gs*Xyl, *Sr*Xyl, and *Bp*Xyl were aligned with *Ec*Xyl43 sequence, and the loops, auxiliary domain, catalytic residues, and active‐site residues are highlighted. The calcium binding site residues are shown in green triangles. (B) Structural alignment of the aforementioned enzymes. *Ec*Xyl43 F507A is depicted in cyan, *Gs*Xyl in green, *Sr*Xyl in brown, and *Bp*Xyl in yellow. The loop containing F507A mutation is highlighted in a black dashed circle. (C) Zoom in the active site area, showing the distances between F506 and E186, and A507 and E186, and the side chains of amino acid residues in the correspondent positions of the superposed enzymes. Figures were generated with ESPript 3 and VMD.

Fig. 3 indicates that, although the overall conformation of the 502–510 loop is conserved among GH43 enzymes, the F507A mutation in EcXyl43 induces a distinct local rearrangement that alters the spatial relationship between this loop and the catalytic tunnel. In comparison with the WT, the mutant F507A shows a dislocation of 2.1 Å of F506 toward the catalytic tunnel (Fig. 6). This effect is quantitatively detailed in Table 4, which reports atom‐specific distances between residues in the loop and the catalytic residue E186 (or its equivalent in homologs). In wild‐type homologous enzymes, the phenylalanine corresponding to F507 in EcXyl43 is positioned at a relatively conserved distance from the catalytic glutamate. When measured from the aromatic CZ atom to the CD atom of the catalytic residue, this distance is approximately 7.8 Å in GsXyl, 8.2 Å in SrXyl, and 8.6 Å in BpXyl, reflecting a common spatial organization that maintains the loop at a defined separation from the active site. In contrast, in the EcXyl43 F507A mutant, the distance between A507 (CB atom) and E186 (CD atom) increases markedly to 12.3 Å, indicating that the mutation displaces this position away from the catalytic center. Concomitantly, the preceding residue in the loop (F506 in EcXyl43) shifts closer to the active site. In EcXyl43 F507A, the distance between F506 (CZ atom) and E186 (CD atom) is reduced to 6.5 Å, which is shorter than the corresponding distances observed for the equivalent residues in other GH43 enzymes. In homologs, these preceding residues—A505 in GsXyl, F507 in SrXyl, and F503 in BpXyl—are typically positioned further from the catalytic residue, with CB–CD distances ranging from ~12 to 14 Å and CZ–CD distances around 9–10 Å (Table 4). Together, these measurements suggests that the F507A mutation does not simply remove an aromatic side chain but triggers a redistribution of spatial constraints within the 502–510 loop. The loss of the bulky phenylalanine at position 507 is compensated by a displacement of F506 toward the catalytic glutamate, effectively shifting the loop closer to the catalytic tunnel and contributing to its local narrowing. This rearrangement provides a structural basis for the altered geometry of the active‐site entrance observed in the mutant structure. (Fig. 4).

**Table 4:** Distances between different atoms in *Ec*Xyl43, *Gs*Xyl, *Sr*Xyl, and *Bp*Xyl.

Enzyme	Residue 1	Atom 1	Residue 2	Atom 2	Distance (Å)
*Ec*Xyl43	F506	CZ	E186	CD	6.5
	A507	CB		CD	12.3
*Gs*Xyl	A505	CB	E187	CD	13.8
	F506	CZ		CD	7.8
*Sr*Xyl	F507	CB CZ	E186	CD CD	12 9.8
	F508	CB CZ	E186	CD CD	10.5 8.2
*Bp*Xyl	F503	CB CZ	Q186	CD CD	13.0 9.6
	F504	CB CZ		CD CD	10.5 8.6

**Fig. 4:**
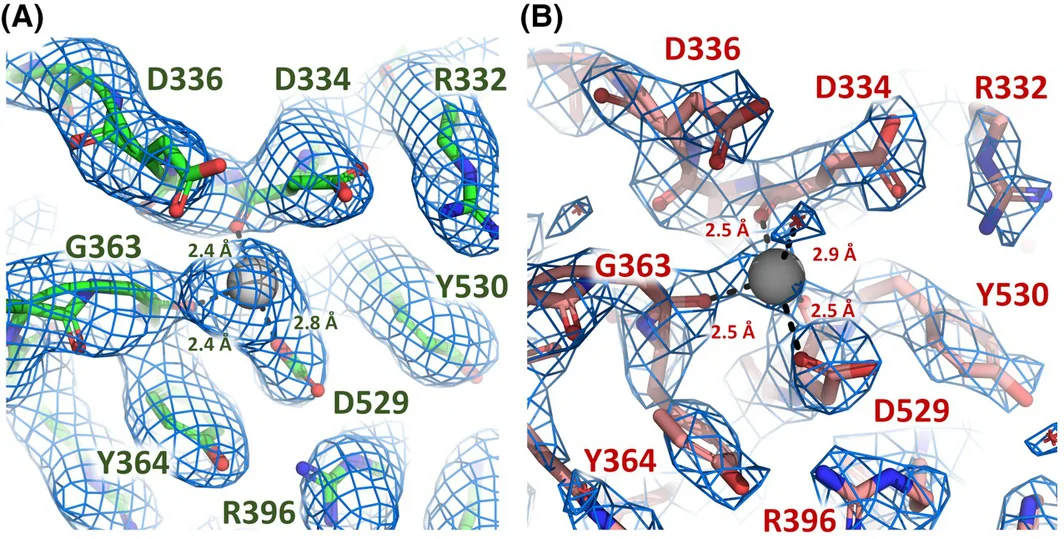
Comparison of the calcium‐binding site in *Ec*Xyl43 WT cryo‐EM and F507A crystal structures. (A) Close‐up view of the calcium‐binding site in the WT cryo‐EM structure. The residues coordinating the Ca^2+^ ion are shown as green sticks. The real‐space density map is displayed as a blue mesh contoured at a level of 2.55 σ. (B) Corresponding view of the calcium‐binding site in the F507A crystal structure. The 2F o–Fc electron density map is shown as a blue mesh contoured at 1.0 σ. In both panels, the calcium ion is represented as a gray sphere. Residues involved in calcium coordination are displayed as sticks, and interatomic distances between the Ca^2+^ ion and coordinating atoms are indicated in Å. Figures were generated with PyMol.

### Role of active site residues and their interactions with the substrate

*Ec*Xyl43 follows the inverting mechanism of enzymatic catalysis, with Glu186 serving as a general acid and Asp14 acting as a general base [26]. In this mechanism, the general base coordinates a water molecule that attacks the glycon C1′ atom, while the general acid protonates the leaving group at the glycosidic oxygen, releasing a sugar monomer as the product [27, 28]. Understanding the interactions between active site residues and the substrate is therefore essential.

To investigate this, we performed a structural superimposition of *Gs*Xyl (PDB code 2EXJ) and *Ec*Xyl43, revealing the distances between key residues and the substrate (Fig. 5). This allowed us to infer potential enzyme‐substrate interactions, as well as residue interactions in the *Ec*Xyl43 F507A mutant.

**Fig. 5:**
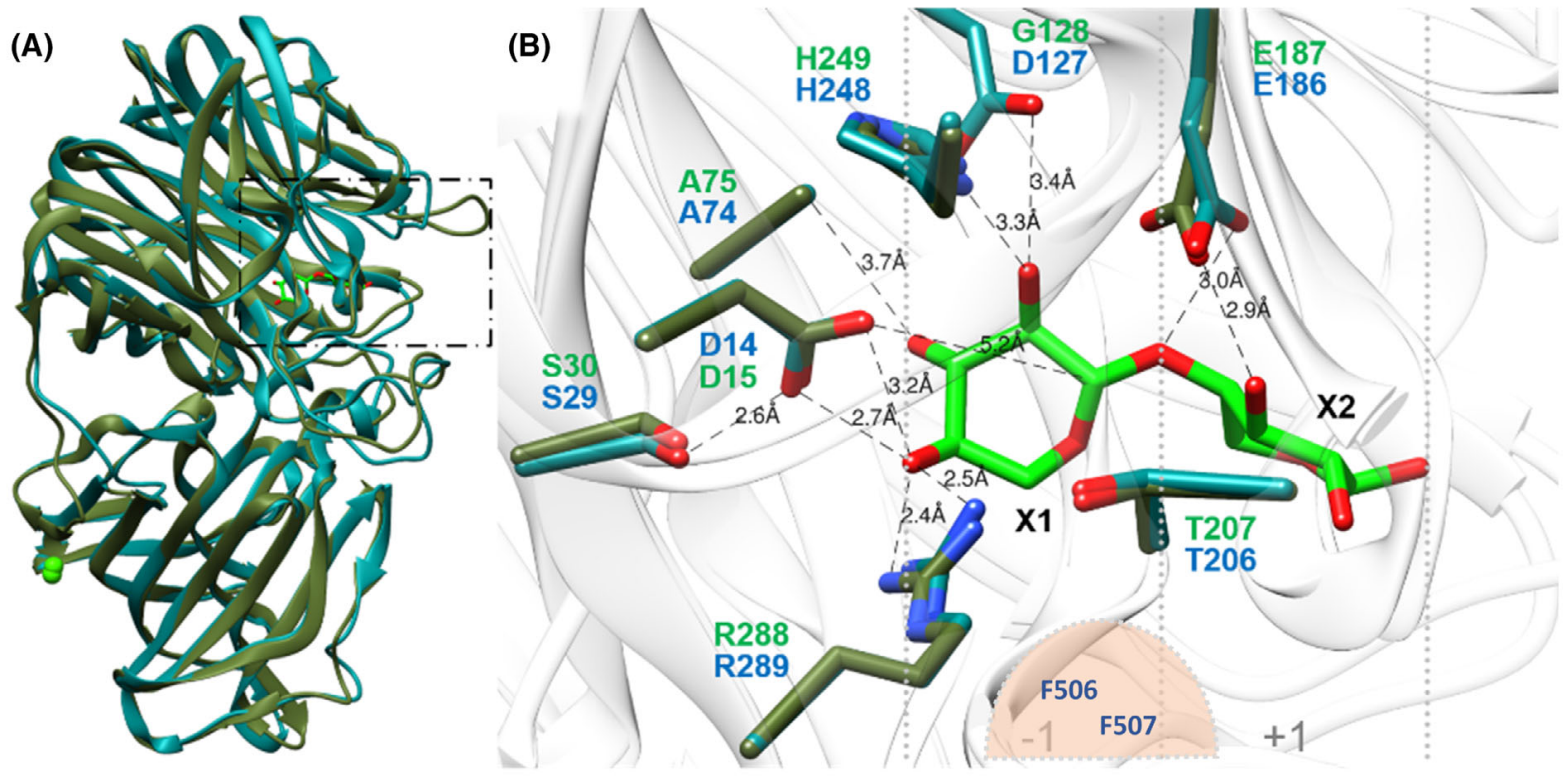
Superimposition of *Ec*Xyl43 F507A and *Gs*Xyl. (A) Superimposition of *Ec*Xyl43 (blue) and *Gs*Xyl (green), with xylobiose from *Gs*Xyl structure in the active site (dashed square). Green spheres represent calcium ions in both enzymes. (B) Zoom of the active site (dashed square in (A)), with the residues (green for *Gs*Xyl and blue for *Ec*Xyl43) and their distances to the ligand atoms. Figures were generated with VMD.

The general acid, Glu186 in *Ec*Xyl43, forms two hydrogen bonds with the substrate. First, it is 3.0 Å from the glycosidic oxygen between subsites −1 and 1, facilitating catalytic activity. Additionally, Glu186 is 2.9 Å from O2 of subsite +1, stabilizing the substrate at this site.

Asp14, a critical residue involved in catalysis as a general base, is expected to lose enzymatic activity when mutated. Indeed, the D14A mutant shows no activity with native substrates [17], as Asp14 activates a water molecule for a single‐displacement attack on the anomeric carbon, essential for glycosidic bond cleavage [24]. The average distance between the oxygen atoms of Glu186 and Asp14 is 8.0 Å, consistent with inverting enzymes (range 8.0–10.0 Å) [26]. The distance from Asp14 (OD1) to the anomeric carbon is 5.2 Å, typical for its role as a general base, while its 2.7‐Å distance to O4 of the xylosidic residue at subsite −1 further supports its catalytic role. Arg289 and Asp127 also interact with the substrate at subsite −1, with distances of 2.5 Å and 3.4 Å, respectively.

Asp127, another key residue, acts as a pKa modulator in GH43 enzymes, influencing the positioning of Glu186 relative to the substrate [29]. At the enzyme's pH optimum of 6.5, Glu186 would typically be deprotonated due to its low pKa of 4.25. Asp127, located 4.4 Å from Glu186, raises the pKa of Glu186, ensuring its protonation at higher pH, which is crucial for its role in catalysis. In GsXyl, the presence of Asp127 shifts the pKa of Glu186 to 7.1 [11].

### Molecular docking of X6 and the active site of *Ec*Xyl43

In order to better understand the higher affinity of *Ec*Xyl43 for longer XOS (with a degree of polymerization up to 6), molecular docking of X6 was performed (Fig. 6). As expected, the interactions between the substrate and the enzyme within subsites −1 and +1 are identical to those observed for X2. In addition, new interactions are established in subsite +2, including hydrogen bonds with Lys99 and His150. Lys99 is highly conserved among GH43 exo‐β‐xylosidases, indicating that this interaction likely represents a general and functionally important feature of substrate recognition in this enzyme family. In contrast, His150 is not strictly conserved, suggesting that this residue may contribute to fine‐tuning substrate binding and specificity in *Ec*Xyl43 rather than representing a universal interaction site. *Ec*Xyl43 interacts with the substrate xylopyranosides at the positions −1, +1, and +2. For sugar residues at positions +4 and +5, no interaction was observed in the initial docking model. The results from docking indicated that X6 bound preferentially to the active site (Fig. 6) –15 out of 24 poses. However, the same parameters used for F507A mutant docking result in only one pose inside the active site out of 24 (Fig. 6). This corroborates our suggestion that F506 may adopt a different conformation when A507 is present, as can be observed in Fig. 6C in comparison with Fig. 6D.

**Fig. 6:**
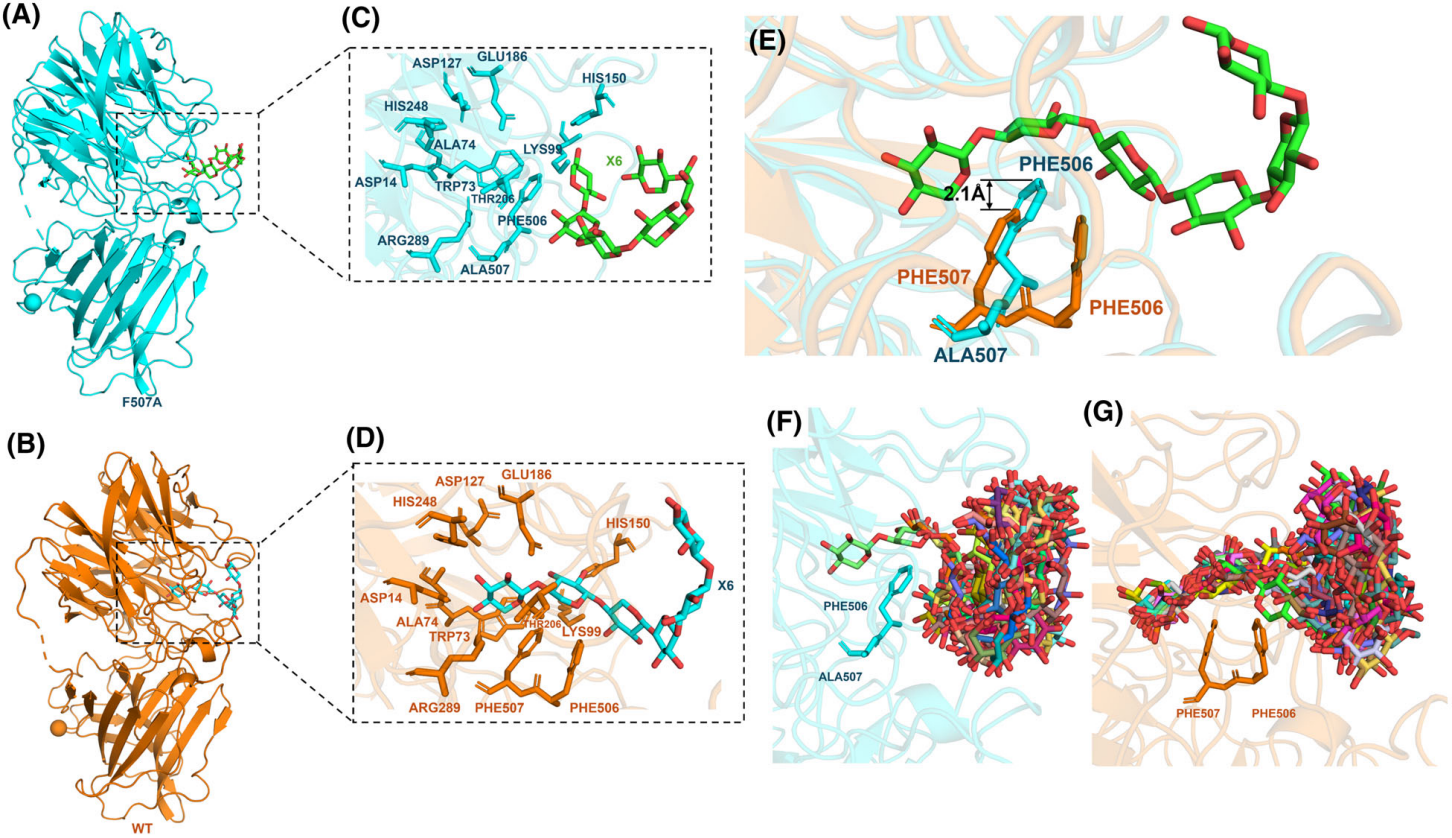
Molecular docking of X6 in the active site of *Ec*Xyl43 WT and F507A mutant. (A) Side view of *Ec*Xyl43 F507A and wild‐type (B) with X6 in its active site (dashed square). (C) and (D) Zoom of active site, showing interaction distances between enzyme and substrate. (E) Superposition of *Ec*Xyl43 WT and F507 with docked xylohexaose, showing F506 closer to the catalytic tunnel in the mutant compared with the wild‐type structure. Docking of xylohexaose into *Ec*Xyl43 F507A (F) and WT (G). Figures were generated with VMD.

Two other highly conserved residues among GH43 family members are Trp73 and Phe507. Trp73 is oriented parallel to the first xylose residue (subsite −1) and stabilizes this xylose unit via van der Waals interactions. It is part of a conserved ^73^WAP^75^ sequence, which forms a pocket to guide substrates to bind in the proximity of catalytic residues [30]. In *Bacillus subitilis* GH43 arabinanase, mutation of Trp74 into alanine affects negatively catalytic activity, and for *Ec*Xyl43, mutation W73G completely abolishes the catalytic activity [17]. At the same time, residue Phe507 is not a catalytic residue and does not belong to the catalytic domain. It is in the loop 502–510 of the ancillary domain that extends within the fivefold beta propeller domain and encloses the active site tunnel. Nevertheless, Phe507 is likely to play a crucial role in the catalytic site, once mutation of the equivalent Phe503 into Tyr in *Bp*XynB enzyme resulted in tighter binding of the second (or aglycon) sugar, promoting reverse reaction of the hydrolase [31]. Furthermore, the mutation of Phe507 to alanine in *Ec*Xyl43 molecular settings completely abrogated its enzymatic activity [17].

### Molecular dynamics simulation of *Ec*Xyl43

To better understand EcXyl43 interactions with ligands, we performed Molecular Dynamics (MD) simulations with X6 docked into the enzyme. The simulations revealed that X6 remains bent over the enzyme surface for a significant portion of the simulation time (~60%), forming additional stabilizing interactions. These interactions provide an enthalpic contribution that stabilizes X6's conformation, reduces its molecular mobility, and shields it from solvent exposure. This stabilization likely explains why EcXyl43 preferentially acts on longer XOS, such as X6, as compared to shorter oligosaccharides (Fig. 6).

### Why F507A mutation abrogates *Ec*Xyl43 enzymatic activity

Our structural analysis showed that the tunnel entrance is significantly narrower in *Ec*Xyl43 F507A mutant as compared to GH43 β‐xylosidases from *G. stearothermophilus* (PDB id: 2EXH), potentially interfering with the access of the substrate to the *Ec*Xyl43 active site (Fig. 7).

**Fig. 7:**
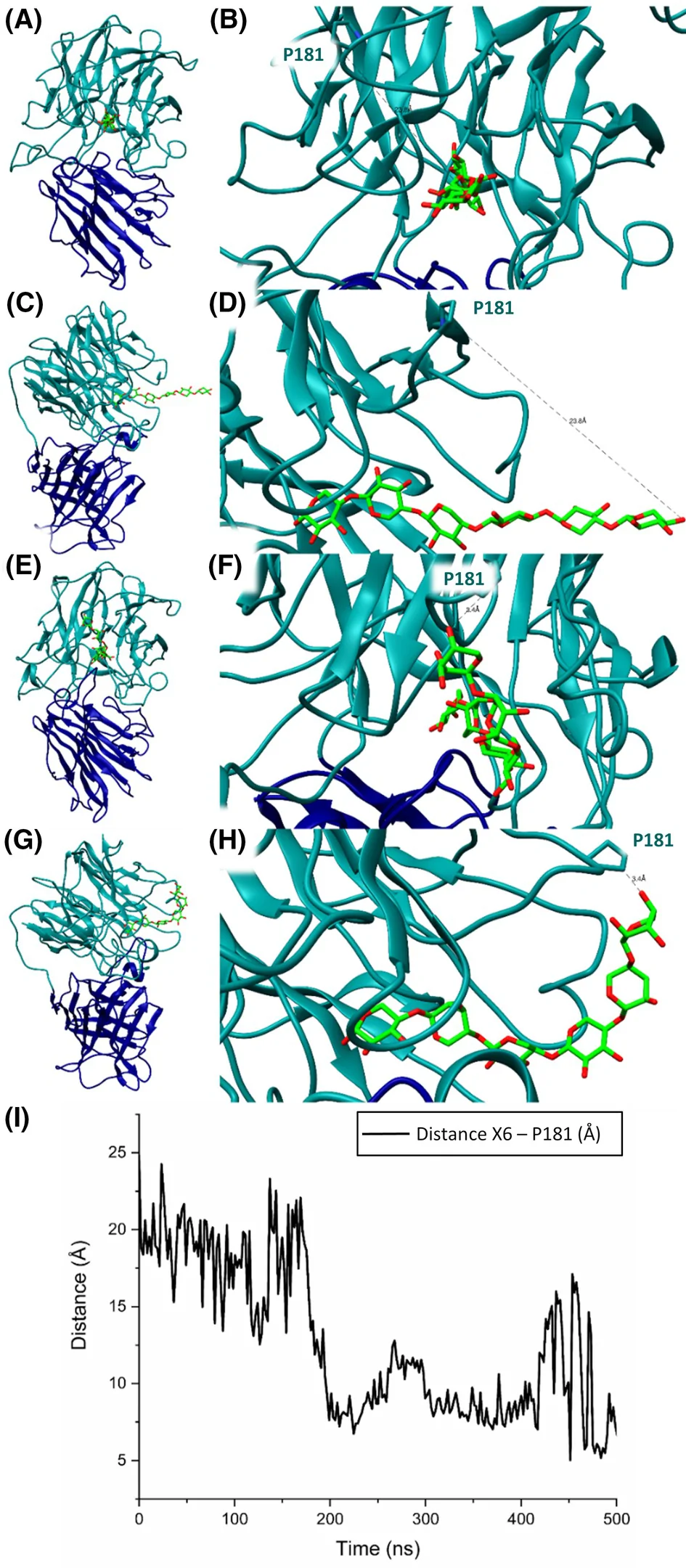
Molecular dynamics of *Ec*Xyl43 with X6 docked in the active site. (A) Initial position of *Ec*Xyl43 and X6 in active site, perpendicular to the enzyme (front view). (B) Zoom of (A), showing the distance of the last xylosidic residue (X6) to H189 (23.8 Å). (C) Same as (A), with 90‐degree rotation along y axis (side view). (D) Zoom of (C), showing the distance from X6 to H189. (E) Intermediate position of *Ec*Xyl43 with X6 in active site, showing the ligand bending toward the loop. (F) Zoom of (E), showing the distance of the last xylosidic residue (X6) to H189 (3.4 Å). (G) Side view of (E), with 90‐degree rotation along y axis. (H) Zoom of (G). (I) Graphic of the distance between last xylosidic residue (X6) to H189 along the simulation (500 ns). Figures were generated with VMD and ChimeraX.

Indeed, the catalytic tunnel of *Ec*Xyl43 is narrower not only as compared to *Gs*Xyl, but also *Sr*Xyl and *Bp*Xyl Fig. 6 (B, D, F, and H). The loop where F507 is located has a characteristic signature sequence ^505^GFF^507^ (Gly505 – Phe506 – Phe507) in *Ec*Xyl43, and Phe507 is mutated; the entire loop shifts toward the catalytic tunnel. Preceding F506 moves toward the active site residues, straightening up the active site and interfering with the ligand binding position (Fig. 7I).

For detailed comparison between the different tunnels of GH43 members with β‐xylosidase activity, *Ec*Xyl43 and *Gs*Xyl43 structures were submitted to PDBsum [32]. The results were analyzed and can be seen in Table 5.

**Table 5:** Tunnel analysis of GH43s with β‐Xylosidase activity. AlR, Aliphatic Residues; ArR, Aromatic Residues; FR, Free radius of tunnel bottleneck (Å) – no sidechain; Hpa, Hydropathy Index (−4.5 hydrophilic to +4.5 hydrophobic); Hph, Hydrophobicity Scale (−1.140 hydrophilic to +1.810 hydrophobic); Len., Length of the tunnel (Å); Lig, Ligand; NeR, Neutral Residues; NR, Negative Residues; Pol, Average of lining aa. polarities (A, G – non‐polar – 0 to polar (S – 1.67) to charged (E – 49.90)); PR, Positive Residues; Rad, Radius of tunnel bottleneck (Å).

Enzyme	Rad	FR	Len.	HPa	HPh	Pol	PR	NR	NeR	AlR	ArR	Lig
*Ec*Xyl43 F507A	1.66	1.88	18.7	−1.34	0.41	24.5	5	2	0	0	5	GOL
*Gs*Xyl	1.55	3.43	16.8	−2.46	−0.34	23.6	5	2	2	0	2	
*Bp*Xyl	2.05	2.54	15.9	0.15	0.45	10.8	2	2	1	1	5	XYP

PDBSum results indicate that *Ec*Xyl43 F507A indeed has a considerably narrower tunnel than *Gs*Xyl, comparing a free radius of tunnel bottlenecks (FR), which have values of 1.88 Å and 3.43 Å, respectively. *Bp*Xyl also has a wider tunnel bottleneck than *Ec*Xyl43 (2.54 Å), indicating that even with the presence of the ligand (xylobiose for *Bp*Xyl), the tunnel shaped by phenylalanine residues in positions equivalent to Phe 507 in *Ec*Xyl43 structure (Phe 506 in *Gs*Xyl and Phe 504 in *Bp*Xyl) is wider than a tunnel of *Ec*Xyl43 with F507A mutation. This sheds light on the structural determinants of the lack of enzymatic activity for *Ec*Xyl43 F507A mutant.

### Molecular dynamics simulations for F507A mutant

In order to further explore the role of residues Phe507 in *Ec*Xyl43 and Ala507 in F507A mutant and their role in active site tunnel structure, three molecular systems were generated and analyzed: *Ec*Xyl43 in apo form, *Ec*Xyl43 with X6 docked in active site and *Ec*Xyl43 mutant F507A in apo form. The RMSD and RMSF in the process of conducted MD simulations are given in Fig. 8.

**Fig. 8:**
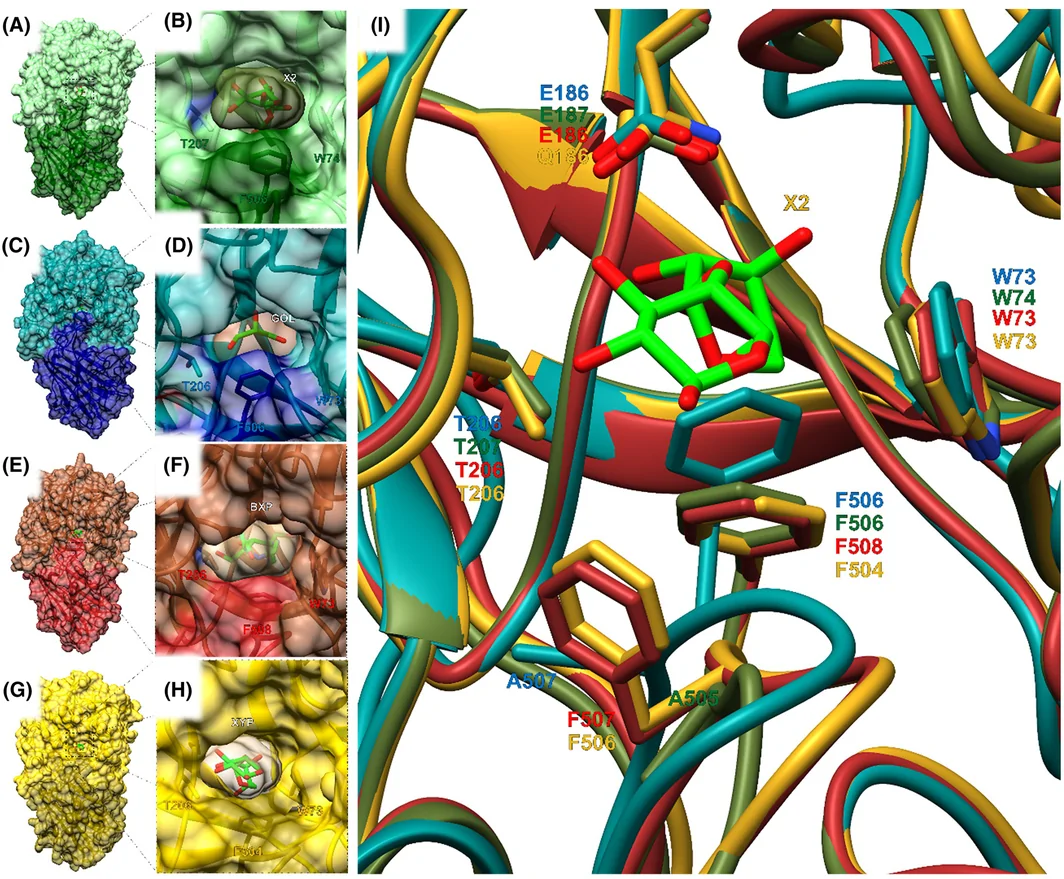
Structural comparison of catalytic clefts. (A) GsXyl (PDB ID: 2EXJ) in light green (catalytic domain) and dark green (ancillary domain), with xylobiose in active site. (B) Zoom of active site, showing the active site cavity with the ligand inside. (C) EcXyl43 in light blue (catalytic domain) and dark blue (ancillary domain), with glycerol in active site. (D) Zoom of active site, showing the ligand bound to the active site. (E) SrXyl (PDB ID: 3C2U) in ruby (catalytic domain) and red (auxiliary domain), with BXP bound to the active site. (F) Zoom into the active site, displaying the ligand bound to the active site. (G) BpXyl (PDB ID: 5ZQX) in yellow (catalytic domain) and gold (ancillary domain), with X2 in the active site. (H) Zoom into the active site, showing the ligand bound to the enzyme. (I) Superimposition of GsXyl (green), EcXyl43 (light blue), SrXyl (red) and BpXyl (yellow) with xylobiose in the active site. Figures were generated with VMD.

Interestingly, the presence of a substrate in the active site does not result in a reduction of the RMSD compared to the initial (experimental) structure (Fig. 8D). This behavior is consistent with the exo‐acting nature of EcXyl43, whose active site forms a narrow tunnel that accommodates only a limited number of xylopyranosidic units (typically up to three). As a consequence, substrate binding involves relatively few enzyme–substrate interactions and is not expected to induce large global conformational rearrangements. In contrast, β‐xylanases from the GH10 and GH11 families are endo‐xylanases and possess wider and more open catalytic clefts, containing multiple subsites that allow binding of longer polysaccharide segments. These extended binding surfaces promote a larger number of enzyme–substrate interactions and can therefore be associated with more pronounced structural stabilization and, in some cases, reduced RMSD values upon substrate binding.

In the opposite direction, an root mean square fluctuation (RMSF) analysis (Fig. 8E) indicates that some residues have higher fluctuation for the *Ec*Xyl43 structure bound to the ligand, as compared to the free enzyme. These results, shown in Fig. 8A–C, suggest that the loops of *Ec*Xyl43, in particular the ones in contact with the solvent, have high mobility. Since loop 502–510, which contains F507, is of particular interest for our study, the RMSF of the region was analyzed in detail (Fig. 8F). Our analysis demonstrates that F507 shows higher RMSF in this region, suggesting that the loop moves much more in order to accommodate the substrate. It can be noticed that the RMSF difference between apo F507A and the apo native form of *Ec*Xyl43 is much higher in the apo form of mutant F507A. It may suggest that mutation F507A may decrease this loop motility.

After RMSF analysis, pairs of distances were analyzed along *Ec*Xyl43 MD trajectories. In order to monitor the tunnel size, two distances were measured in two systems: F507–E186 in the native *Ec*Xyl43, and A507–E186 in the *Ec*Xyl43 F507A mutant (Fig. 9). Taken together, these analyses indicate that substrate binding to EcXyl43 does not promote major global conformational stabilization but instead is accommodated through local and dynamic rearrangements of flexible loops surrounding the catalytic tunnel. In particular, the 502–510 loop displays enhanced mobility upon substrate binding, with F507 playing a central role in modulating tunnel geometry and accessibility. The F507A mutation might alter this dynamic behavior, reducing loop flexibility and perturbing the delicate balance between tunnel openness and adaptability that is required for productive substrate accommodation. These results highlight that, in exo‐acting GH43 enzymes, catalytic efficiency is governed not only by static structural features but also by finely tuned loop dynamics that regulate substrate entry and positioning.

**Fig. 9:**
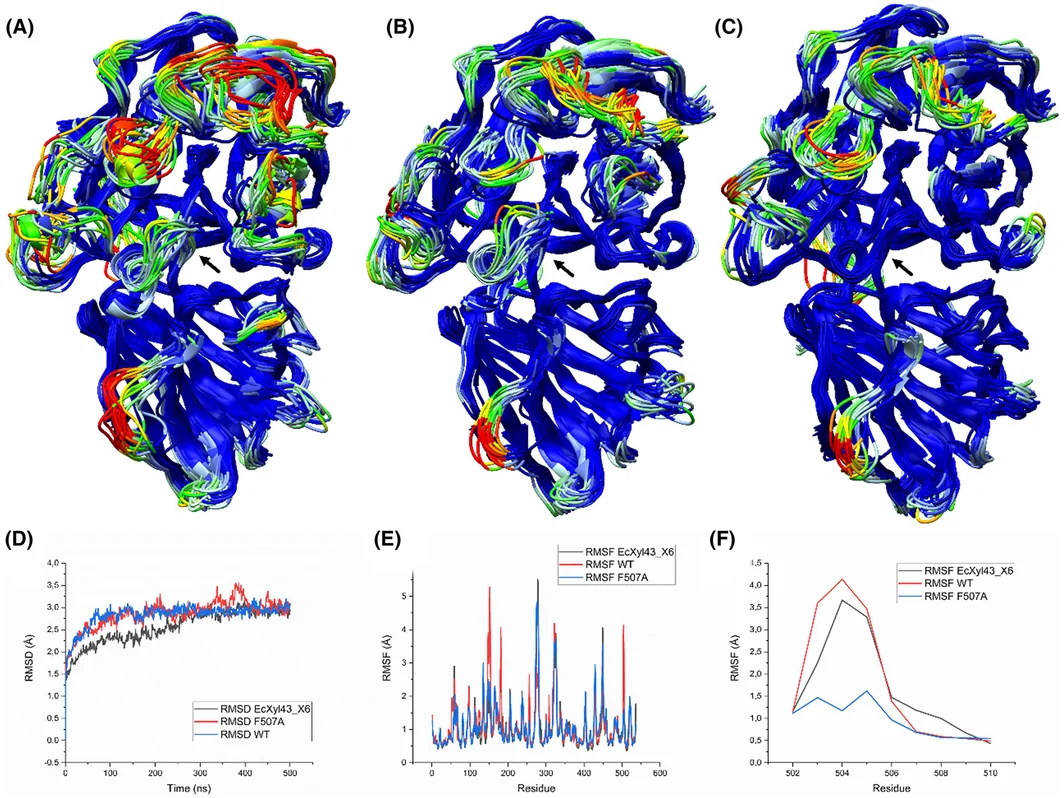
Trajectories of *Ec*Xyl43 along Molecular Dynamics. Cluster representation of *Ec*Xyl43 with X6 docked in active site (A), *Ec*Xyl43 in Apo form (B), *Ec*Xyl43 F507A in apo form (C) over 500 ns MD simulation with 1 ns interval, with structures colored by RMSF from blue (minimum) to red (> 3 Å). The arrows indicate the loop 502–510, containing Phe507. The figures were produced with mdlovofit [33]. (D) RMSD of the systems along the dynamic simulations. (E) RMSF of the systems along the molecular dynamics, per residue. (F) RMSF of the systems, comparing loop region of residues 502–510 (where mutation F507A is located). Figures were generated with VMD.

## Material and methods

### Cloning, expression and purification

Gene cloning and protein expression was previously described in Ontañon *et al*. [17]. *Escherichia coli* strain DH5α was used as a recipient for transformations during plasmid construction and plasmid propagation and storage, while *E. coli* Rosetta (DE3) (Novagen, Darmstadt, Germany) was used for protein expression.

Briefly, a preculture of *E. coli* BL21‐pET28a *Ec*Xyl43 was started in LB‐kanamycin (50 μg·mL^−1^) and incubated at 37 °C and 200 rpm in a shaker, overnight. A proportion was 5 mL of preculture for 500 mL of expression was used. The expression was carried out at 37 °C and 200 rpm in a shaker for approximately 4 h until the cell density reached an OD_600_ of 0.6. Then, the grown cells were induced with 1 mm isopropyl β‐D‐thiogalactopyranoside (IPTG) at 37 °C under continuous agitation in a shaker at 200 rpm for 16 h. The culture was centrifuged at 4000 ***g*** at 4 °C for 20 min and the cell pellet was resuspended in a lysis buffer, pH 8.0 (300 mm NaCl, 50 mm NaH_2_PO_4_ and 10 mm imidazole). The resuspended cells were disrupted by two cycles of freezing in liquid nitrogen followed by thawing under room temperature. Then lysozyme was added, on ice so that the final concentration was 1.0 mg·mL^−1^. After 30 min, six 10‐s cycles of sonication on ice bath using a 550 Sonic Dismembrator Sonifier (Fisher Scientific, Hampton, USA) at 28% amplitude were performed. The lysate was clarified by centrifugation at 10,000 ***g*** for 30 min at 4 °C to remove cell debris and the supernatant was used for *Ec*Xyl43 purification.

The supernatant was applied to a 5 mL Ni‐NTA Superflow column (Qiagen, Hilden, Germany), previously equilibrated with the lysis buffer. Three volumes of wash buffer pH8 (300 mm NaCl, 50 mm NaH_2_PO_4_ and 20 mm imidazole) were used to wash the column. The protein was eluted with two elution buffers pH8, both with 300 mm NaCl and 50 mm NaH_2_PO_4_, but with different imidazole concentrations: The first buffer had 250 mm while the second had 500 mm. Then protein fractions were pooled and concentrated in a 50 kDa molecular cutoff concentrator to reduce imidazole concentration to 20 mm. Purification was evaluated by 15% sodium dodecyl sulfate gel electrophoresis (SDS/PAGE) analysis under denaturing conditions and subsequently stained with Coomassie Brilliant Blue R‐250. The purified enzyme was further concentrated using a 10 kDa Vivaspin Concentrator (GE‐Healthcare) at 1500 × ***g*** and its concentration was determined by measuring absorption at 280 nm using Nanodrop 1000 spectrophotometer (Thermo Scientific, Waltham, EUA) and applying the calculated extinction coefficient (*Ɛ* = 150 690·M^−1^·cm^−1^). Final yield was 2 mg of pure protein per liter of culture. This methodology was also used for expression and purification of D14A and F507A mutants.

### Enzymatic assays

Enzymatic activity was carried out using 2.5 mm 4‐nitrophenyl β‐D‐xylopyranoside (*p*NPX) as a substrate. Reactions were carried out in 50 mm NaH_2_PO_4_, pH 6 with 0.04 mg·mL^−1^ enzyme for 10 min (100 μL total volume) and stopped with 0.5 mL of 2% Na_2_CO_3_. The μmol of *p*‐nitrophenol (pN) released was determined by absorbance at 410 nm with a pN curve as standard reference. The enzymatic assay was conducted in triplicates accompanied by a control measured in the absence of GH43 enzyme.

### Cryo‐EM data collection and processing

For grid preparation, freshly purified *Ec*Xyl43 samples were thawed in ice, spined, and diluted in gel filtration buffer. Then, 3 μL of 0.25 mg·ml^−1^ of samples was applied on glow discharged Quantifoil 200 mesh Cu R2/2 grid. Grid was blotted using Vitrobot Mark IV (FEI) for 2.5 s with blot force 1 at humidity 100% and 4 °C before plunge freezing into liquid ethane.

Images were collected in movie‐mode using a Titan Krios (ThermoFisher Scientific) equipped with a Cs corrector and using a Falcon 3EC camera (ThermoFisher Scientific) operated in counting mode. In total, 10 176 movies were recorded with a pixel size of 2.46 Å and at targeted defocus of −1.5 and −2.0 μm. The total exposure dose of 30.0 e^−^/ Å^2^ was split over 20 frames.

Data processing was performed using CryoSPARC v4.1.2 [34]. The initial 10 176 movies were aligned with Patch Motion Correction, and their contrast transfer function (CTF) was estimated using the Patch CTF Estimation tool. After multiple rounds of manual picking and classification, a subset of particles was selected to train Topaz [35], which was then used to pick 1 045 759 particles. These were extracted with a box size of 400 px and binned to 150 px.

Two rounds of 2D classification (*n* = 100) yielded 695 480 particles, which underwent two rounds of Ab‐initio reconstruction and heterogeneous refinement (*n* = 4). A final subset of 438 836 particles was re‐extracted at full resolution (400 px) and subjected to homogeneous refinement, nonuniform refinement, and local refinement, all with D2 symmetry imposed. The final model achieved a gold‐standard FSC resolution of 2.65 Å. Model building was conducted using the initial AlphaFold 2.0 [36] model refined in real space using Phenix Real Space refinement and Coot [37, 38]. Protein was deposited in the Protein Data Bank (PDB) under the code 8UWS, while aligned movies are available in EMPIAR under the code EMPIAR‐11768. Details of the data processing pipeline are available in Fig. 1, and data parameters and statistics are available in Table 1.

### Crystallization, data collection, and processing *Ec*Xyl43 F507A

For crystallization assays, the purified *Ec*Xyl43 and its catalytically inactive mutant *Ec*Xyl43 F507A were concentrated to 10 mg·mL^−1^. The crystallization trials were set using a crystallization robot Honeybee (DIGILAB, Massachusetts, USA), and the crystals were grown by the sitting‐drop vapor‐diffusion technique with different protein‐to‐mother liquor ratios, using Crystal Screen kit (Hampton Research, California, USA). The crystals of *Ec*Xyl43 F507A were detected under crystallization conditions consisting of 4 M sodium formate, pH 8.0 at 20 °C, with 2 μL of protein mixed with 1 μL of buffer in the crystallization drop. Despite continuous efforts, we were unable to crystallize WT *Ec*Xyl43.

The resulting *Ec*Xyl43 F507A crystals were flash‐frozen in liquid nitrogen after bathing in Glycerol (30% v/v). X‐ray diffraction data were collected at the W01B‐MX2 beamline of the Brazilian National Synchrotron Light Laboratory (Campinas, Brazil) [39], using a Pilatus2M detector (Dectris, Philadelphia, USA), with an exposure time of 2 s per image and an oscillation angle of 0.2°. The diffraction image data were indexed, integrated using XDS package [40] and scaled with AIMLESS [39].

Molecular replacement was performed using Phaser [41] with the atomic coordinates of *Gs*Xyl43 (57% sequence identity) as a search model. The resulting structure was then submitted by Autobuild [42], and refinement cycles were performed using Coot [38] and PHENIX‐refine [43]. Model quality was evaluated using Molprobity [44]. The calcium ion binding sites were confirmed using CheckMyMetal [45]. The atomic coordinates have been deposited with the Protein Data Bank (PDB) under the accession code 7K1R. Data collection statistics are available in Table 2.

### Structural analysis

*Ec*Xyl43 sequence was submitted to BLASTP [46], using Protein Data Bank (PDB) as a database. The top result was GH43 from *Geobacillus stearothermophilus* (*Gs*Xyl), with identity value of 56.45% and query cover of 99%. For structural comparison, *Ec*Xyl43 F507A crystallographic structure was submitted to DALI [47] to compare with other 3D structures in PDB database. The top three enzymes with β‐xylosidase activity coincided with top results in BLASTP search and were selected for further analysis. Three GH43 amino acid sequences from *Geobacillus stearothermophilus*, *Bacillus pumilus*, and *Selenomonas ruminantium* (PDB accession codes 2EXH_A, 5ZQX, and 3C2U, respectively) were aligned against *Ec*Xyl43 in Clustal Omega [48] and then submitted to ESPRIPT [49]. Structural figures were made with Pymol (The PyMOL Molecular Graphics System, version 1.2r3pre; Schrödinger, LLC, Cambridge, MA, USA) or ChimeraX [50]. For structural comparison purposes, *Ec*Xyl43 F507A crystal structure was superimposed with GH43 β‐xylosidases from *G. stearothermophilus* (*Gs*Xyl, PDB code 2EXH) and *Gs*Xyl complexed with xylobiose (PDB code 2EXJ).

### Molecular docking

In order to understand the molecular interactions between *Ec*Xyl43 and XOS, a xylohexaose (X6) molecule was docked into *Ec*Xyl43 using the program DockThor [51]. Briefly, the coordinates for the substrates were generated using the GLYCAM web server [52] and the resulting file was used in DockThor along with crystal and Cryo‐EM structures of *Ec*Xyl43 F507A and WT, respectively. The coordinates (x,y,z) used for docking were the same as those from the glycerol molecule (active site in F507A). The poses were analyzed using PyMOL and Chimera [50].

### Molecular dynamics simulations

The molecular dynamics simulations of *Ec*Xyl43 were carried out using NAMD [53], adapting the protocol from Kadowaki *et al*. [54]. The protonation states at pH 6.5 and in the presence of 150 mm NaCl system were estimated using H++ server [55]. The simulations were set up using the CHARMM‐GUI for system preparation [56], and CHARMM36 carbohydrate force field to describe xylohexaose [57]. The enzyme–carbohydrate structures were solvated in cubic boxes with a solvent layer at least 10 Å thick, with sodium and chloride ions being added to reach neutrality. Each system was minimized and heated accordingly following the protocol. The MD simulations were performed with NAMD in the NVT ensemble for 100 ns with a 2‐fs time step at 310 K. The MD trajectories were analyzed using VMD [58], mdlovofit [59], and computational codes programmed in‐house.

## Author contributions

LB was involved in conceptualization, methodology, investigation, visualization, and writing. ASG was involved in conceptualization, methodology, investigation, visualization, and writing. CC was involved in methodology, investigation, and writing. VP was involved in methodology, investigation, and writing. RSF and OMO were involved in conceptualization, methodology, and writing. EC was involved in conceptualization, methodology, writing, and funding acquisition. IP was involved in conceptualization, project administration, writing and funding acquisition.

## Conflict of interest

ASG is an employe of Echo Life Sciences. Other authors declare no conflict of interest.

## Data Availability

Structure factors and atomic coordinates have been deposited with the protein data bank under accession codes PDB IDs: 8UWS and 7K1R. Electron density maps are available at EMDB under code EMD‐42680. Aligned movies are available at EMPIAR under code EMPIAR‐11768. Other data are available from the corresponding author upon reasonable request.
